# The potential HLA Class I-restricted epitopes derived from LeIF and TSA of *Leishmania donovani* evoke anti-leishmania CD8+ T lymphocyte response

**DOI:** 10.1038/s41598-018-32040-x

**Published:** 2018-09-21

**Authors:** Manas Ranjan Dikhit, Sushmita Das, Vijaya Mahantesh, Akhilesh Kumar, Ashish Kumar Singh, Budheswar Dehury, Ajaya Kumar Rout, Vahab Ali, Ganesh Chandra Sahoo, Roshan Kamal Topno, Krishna Pandey, V. N. R. Das, Sanjiva Bimal, Pradeep Das

**Affiliations:** 1 BioMedical Informatics Division, Rajendra Memorial Research Institute of Medical Sciences, Agamkuan, Patna, 800007 Bihar India; 2 Department of Immunology, Rajendra Memorial Research Institute of Medical Sciences, Agamkuan, Patna, 800007 Bihar India; 3 Department of Microbiology, All India Institute of Medical Sciences, Patna, 801507 Bihar India; 4 BioMedical Informatics Centre, ICMR-Regional Medical Research Centre, Bhubaneswar, 751023 Odisha India; 5 Biotechnology Laboratory, ICAR-Central Inland Fisheries Research Institute, Barrackpore, Kolkata, 700120 West Bengal India; 6 Department of Clinical Biochemistry, Rajendra Memorial Research Institute of Medical Sciences, Agamkuan, Patna, 800007 Bihar India; 7 Department of Epidemiology, Rajendra Memorial Research Institute of Medical Sciences, Agamkuan, Patna, 800007 Bihar India; 8 Department of Clinical Medicine, Rajendra Memorial Research Institute of Medical Sciences, Agamkuan, Patna, 800007 Bihar India; 9 Department of Molecular Parasitology, Rajendra Memorial Research Institute of Medical Sciences, Agamkuan, Patna, 800007 Bihar India

## Abstract

To explore new protective measure against visceral leishmaniasis, reverse vaccinology approach was employed to identify key immunogenic regions which can mediate long-term immunity. In-depth computational analysis revealed nine promiscuous epitopes which can possibly be presented by 46 human leukocyte antigen, thereby broadening the worldwide population up to 94.16%. This is of reasonable significance that most of the epitopes shared 100% sequence homology with other *Leishmania* species and could evoke a common pattern of protective immune response. Transporter associated with antigen processing binding affinity, molecular docking approach followed by dynamics simulation and human leukocyte antigen stabilization assay suggested that the best five optimal set of epitopes bind in between α1 and α2 binding groove with sufficient affinity and stability which allows the translocation of intact epitope to the cell surface. Fascinatingly, the human leukocyte antigen stabilization assay exhibited a modest correlation with the positive immunogenicity score predicted by class I pMHC immunogenicity predictor. A support for this notion came from ELISA and FACS analysis where the epitopes as a cocktail induced CD8+ IFN-γ and Granzyme B levels significantly in treated visceral leishmaniasis subject which suggests the immunogenic ability of the selected epitopes.

## Introduction

The leishmaniases are a complex of protozoan vector-borne diseases which mostly affect and impact “the bottom billion” of people living in poverty with severe clinical and socioeconomic consequences. More than 88 countries have reported cases of leishmaniasis with 0.7–1.2 and 0.2–0.4 million cases reported per annum of cutaneous leishmaniasis (CL) and visceral leishmaniasis (VL), respectively^1^. The most severe and fatal form of this disease is VL which affects cells of the reticulo-endpthelium system of the body (http://www.cdc.gov/NCIDOD/DPD/parasites/leishmania/). Although, several antileishmanial drugs including amphotericin B, paromomycin, and miltefosine are currently in use, they are not fully effective, due to resistance, high toxicity, cost, and different modes of administration^2,3^. Despite a huge number of attempts were made on different vaccination strategies, none of them are in routine use for humans^4,5^. Most human beings who developed leishmaniasis or asymptomatic infection are immune to consequent infections, which make vaccine development rational^6^.

The decisive immune factor that controls of *Leishmania* infection is interferon-γ(IFN-γ) production which not only protects the primary infection but also results in a lifelong immunity to re-infection^7,8^. Although CD4+ T cells are major source of IFN-γ production, CD8+ T-cells are profoundly involved in immune cell activation against *Leishmania* by producing IFN-γ and expressing cytotoxic mediators such as granzyme^9^. Furthermore, the proactive role of CD8+ T cells has been elucidated in the control of *Leishmania major* infection^10^. Another study also suggested that CD8+ T cells purified from *L*. *infantum*-infected mice expressed IFN-γ and tumor necrosis factor alpha (TNF-α)and showed significant cytotoxic activity against the infected cells expressing *Leishmania* antigens^11^. CD8+ T-cells may not merely play a crucial role in immune protection by releasing various cytokines, but they could also be involved in the recruitment of inflammatory cells and in the maintenance of granulomas^12^. Many leishmanial targets have already been identified and vaccination with component proteins, such as P8, gp46, hydrophilic acylated surface protein B1, kinetoplastid membrane protein 11, CPB-Cathepsin L-like protease, CPC-cathepsin B-like protease and protein disulfide isomerase^13–18^ strongly participate CD8+ T-cell-mediated cellular immune activation. Although many of these strategies have resulted in protection in either mouse model or in treated VL subjects, none of them have entered into the clinical trial.

Two candidate antigens i.e thiol-specific antioxidant (TSA) and *Leishmania* eukaryotic initiation factor (LeIF) have been able to elicit relatively protective effects through CD8+ T-cells mediated activity. Some studies, in mice infection, has assessed the protective effects of DNA vaccines containing LeIF and TSA of *Leishmania major* shows that a bivalent vaccine containing two distinct antigens may induce more potent immune responses against tegumentary leishmaniasis^19^. Also, background information on efforts of finding immunogenic epitope in proteins known as virulence factors as prototype vaccine against leishmaniasis, need to be considered^20–23^.

In recent years, the ability of T cells in protection and long-lasting resistance to infection has opened up a new approach in *Leishmania* vaccine development known as “Polytope Vaccine”^24^. Several studies also revealed that the human leukocyte antigen (HLA) restricted epitope-based vaccines strategy seem to be inducing more potent responses than whole antigen vaccines^25,26^. To cope with the HLA diversity, we mined the proteome of TSA and LeIF and included large numbers of different immunogenic epitopes. In this milieu, we hypothesized that the Insilco approach to screen potential epitopes and evaluation of their ability to modulate immune cells would help the search for potential immunogenic epitopes.

## Materials and Methods

### Peptide curation and analysis

The full-length amino acid sequences of TSA (Acc No. AAY88228.1 strain MHOM/SD/001S-2D) and LeIF (Acc. No. XP_003858063.1 strain BPK282A1) were retrieved from National Center for Biotechnology Information (NCBI) protein database. The previous study has already documented these proteins as candidate antigens based on their ability to evoke CD8+ T cells^27–29^. The amino acid sequences were subjected to 9 mer HLA A2 restricted epitope prediction using five different kinds of software: (I) SYFPEITHI^30^, (II) Rankpep^31^, (III) Epijen^32^ and (IV) nHLApred^33^ according to our previously described methodology with certain modifications. The threshold value was adapted from our previously published literature: 22 for SYFPEITHI, 65 for RANKPEP and EpiJen output cut-off set at 5%^3,17,34^. For nHLApred, the default threshold value 0.5% with score 0.5 was considered. The ultimate consensus epitopes were subjected to BLAST search against *Homo sapiens* to avoid the sequence homology^35^. The peptide sequence with 100% query coverage and 100% identity to human proteome were removed from the study.

### Prediction of population coverage and TAP transport efficiency

The HLA polymorphism influences the epitope-binding specificities and therefore HLA cross-presentation ability of epitope play a crucial role to cope with this diversity. To ensure the HLA cross-presentation, selected peptides were further investigated by NetMHCpan3.4^36^. This web server captures differences in the length profile of binders to different HLA allele leading to increased efficiency for probable epitope identification. The theoretical population coverage of promiscuous epitopes was evaluated using IEDB tool (http://tools.immuneepitope.org/tools/population/iedb_input). The amalgamation of HLA binding efficiency with transporter associated with antigen processing(TAP) transport ability augmented the vaccine potential of candidate epitope^37^. In this notion, the TAP binding ability of selected promiscuous epitopes was evaluated by TAPPred^38^.

### Structure prediction of peptides and their binding analysis with the HLA A0201 allele

Now a day, the use of combined structure-sequence-based prediction approach not only enhances the epitope prediction efficiency, but also allows us to analyze docked epitope orientation. The 3D coordinates of the shortlisted epitopes were predicted by PEPFOLD web server^39^. The crystal structure of HLA A0201 protein (PDB ID: 1I4F) coupled with tumor peptide was downloaded from the Protein data bank. This 10-mer antigenic fragment was used as the positive control where as VSV8 peptide (RGYVYQGL) was used as negative control^40^. Docking calculation was performed using PatchDock web server^41^. Twenty high-scored HLA-Peptide complexes were further refined by the FireDock web server^42^.

### Molecular dynamics simulation of the HLA-epitope complexes

To explore the stability and conformational flexibility (global and local) of all the selected HLA-epitope (including the control) systems using GROMACSv5.1 package employing AMBER99SB-ILDN force field parameter and the TIP3P water model. Molecular dynamics (MD) simulations of all the complex (a total of 10) were performed as described previously^7,43^. For each system, a cubic box a minimal distance of 10 Å between the complex and edge of the box, which was then solvated using periodic boundary condition. Each system was neutralized with counter-ions with a strength of 0.15 M Na^+^/Cl^−^. To remove any unfavorable interactions, steepest descents minimization was performed which was followed by two-step equilibrium to generate the starting structures for the production simulations. During equilibration, position restraints were applied to all atoms to avoid any configuration changes. After completion of the two equilibration phases i.e., NVT and NPT, production of MD simulations were conducted for 1000 picoseconds (ps) after taking away the position restraints. Finally, the equilibrated structures were subjected to MD simulations for 20 ns (20000 ps) with linear constrain (LINCS) algorithm. Particle Mesh Ewald (PME) method was used for calculation of long-range electrostatic forces with a grid size of less than 1 Å in all dimensions. The non-boned van der Waals and electrostatic forces were truncated at 12 Å and smoothly switching at 10 Å. Trajectory snapshots were stored at every 0.2 ps during the simulation period, and 3D coordinate files harvested after every 2 ns for post-dynamic analysis. The built-in modules of Gromacs including *gmx rmsd*, *gmx gyrate* and *gmx hbond* were employed to calculate the root mean square deviations (RMSD), radius of gyration (Rg) and intermolecular hydrogen bonds (Hb) between protein and epitope for each system. All 2-D graphs obtained from MD trajectories were plotted using the Xmgrace tool.

### Principal component analysis

Principal Component Analysis (PCA) or Essential Dynamics (ED) is one of the promising approach for unveiling high-amplitude motion in proteins that is based on the eigenvectors (EVs) of the covariance matrix of atomic fluctuations. We employed ED method to calculate the eigenvectors (protein backbone atoms) and eigen values, and their projection along the first two principal components (i.e., PC1 and PC2). The protein molecular segments that are responsible for the most significant collective motions was inferred through PCA using built in modules i.e., *gmx covar* and *gmx anaeig* of GROMACS.

### MM/PBSA binding free energy

The binding free energies of each HLA-epitope complex was calculated using MM/PBSA approach employed in *g_mmpbsa* tool^44^. A total of 200 snapshots extracted from last 10 ns of MD trajectory was employed for estimation binding energies utilizing APBS to solve Possion-Boltzman equations. Following equation was employed to estimate the binding free energies (Δ*G*_*bind*_) of each complex:

$${G}_{bind}={G}_{complex}-(Gprotein+{G}_{peptide})$$

### Prediction of epitope conservancy

The corresponding protein sequences of different *Leishmania* species were downloaded from NCBI protein database. The level of conservancy of the selected epitopes among the *Leishmania* species was evaluated using the epitope conservancy tool (http://tools.immuneepitope.org/tools/conservancy/iedb_input) followed by BLASP analysis against *Leishmania* species (taxid: 5658). The level of similarity was evaluated based on the query coverage and identity against *L*. *donovani*, *L*. *infantum*, *L*. *major*, *L*. *braziliensis*, *L*. *Mexicana* and *L*. *guyanensis* protein sequences. The antigenic properties of candidate epitopes were further evaluated by Kolaskar and Tongaonkar Antigenicity method available in the IEDB^45^. Furthermore, the efficiency of the shortlisted epitopes to provoke an immune response against host was evaluated by T cell class I pMHC immunogenicity predictor tool^46^. To corroborate the specific immune responses induced by the candidate epitopes against the *Leishmania* parasite rather than the host tissue, the ToxinPred web server was used to validate the epitopes’ non-toxicity with default parameters^47^.

### Peptide synthesis

The candidate peptides were synthesized with more than 95% purity by Peptide 2.0 (Chantilly, VA, USA). The lyophilized powder was dissolved in 10% dimethyl sulphoxide or DMSO (Sigma, Steinheim, Germany) and stored aliquoted at −80 °C till use.

### Soluble *Leishmania* Antigen (SLA) preparation

*Leishmania donovani* (Ag83)was cultured in M199 with 10%heat-inactivated fetal bovine serum. Cultured *L*. *donovani* promastigote (250 × 10^6^/ml) in 5 ml of cold sterile phosphate-buffered saline (PBS) was subjected to five cycles of freeze and thaw in −195 °C liquid nitrogen and 37 °C water bath. Then, it was centrifuged at 10,000 g for 20 min at 4 °C^48^. The supernatant containing SLAs were collected and protein concentration was measured by Lowry’s method and stored at −80 °C until further use.

### Sample collection and peripheral blood mononuclear cells isolation

Sixteen successfully treated VL subjects (1 month to 3.5 years after treatment with standard/single dose of Amphotericin B) of both sexes (9 male and 7 female) aged from 19 to 36 years were sampled from the outdoor patient department of Rajendra Memorial Research Institute of Medical Sciences or from the endemic villages. Those treated subjects who were considered to be clinically cured (absence of amastigote confirmed by microscopic/RTPCR) after treatment (1 month after the end of treatment) were enrolled in this study. Each time, 8 ml of blood sample was collected in a 10 ml sterile sodium heparin vacutainer (BD Bioscience San Diego, CA), kept in a refrigerator (4–8 °C) and processed within 5–6 h. Some of the volunteers took part in this study donated blood more than once when needed. Measurement of body temperature, body weight, total and differential WBC count, hemoglobin, blood sugar, serum creatinine, and prothrombin was performed in all cases. The study began after obtaining informed and written consent from the participants. The study was started after obtaining their informed and written consent. The study was approved by and carried out under the guidelines of ethics committee for human studies, Rajendra Memorial Research Institute of Medical Science, Patna, India. The collected blood samples were diluted 1:1 ratio with sterile PBS and Peripheral blood mononuclear cells (PBMCs) were isolated by density centrifugation through Ficoll-Hypaque (Sigma) according to our previously described methodology^49^. A volume of 100 μl PBMCs of each sample was screened for the expression of HLA A2 molecules on the cell surface using a PE-conjugated monoclonal antibody (clone: BB7.2; Pharmingen). Samples were then analyzed directly by FACS Calibur (BD Biosciences). Rest PBMCs were counted, and used within 90 min.

### Peptide-binding assay

The efficiency of the shortlisted epitopes to stabilize the HLA-A 02 allele was measured by HLA stabilization assay using TAP-deficient human cell line that express HLA-A2 (T2 cell line) according to our previously described methodology with certain modifications^49^. Briefly, T2 cells (2 × 10^5^/well) were cultured with 20 μg/mL of individual peptides in serum-free RPMI medium for 20 hours at 37 °C in 5% CO_2_. The HLA stabilization assay was performed in triplicate for each peptide. The tumor HLA A2 bound 10mer peptide was used as positive control. The expression of HLA-A 02 on T2 cells was measured by PE-conjugated mouse anti-human HLA-A 02 monoclonal antibody (clone: BB7.2; Pharmingen), the samples were acquired by FACS Calibur and analyzed by CellQuest software (BD Biosciences). The binding affinity of each peptide was measured by a fluorescence index: FI = (mean PE fluorescence with the given peptide−mean PE fluorescence without peptide)/(mean FITC fluorescence without peptide). The epitope with a fluorescence index (FI) more than 1 was considered as strong binding affinity epitopes^17^.

### Cytokine enzyme-linked immunosorbent assay (ELISA) for total IFN-γ and Granzyme B production

PBMCs (1 × 10^6^/mL) from six HLA A2 positive treated VL subjects were cultured with cocktail of peptide (20 μg/mL) in RPMI medium (Sigma) supplemented with 10% human FBS (Sigma), 1% HEPES (Sigma), 2 mmol/L L. Glutamine (Sigma) and 0.1% Gentamicin (Sigma). The cells were incubated for 72 hrs at 37 °C and 5% CO_2_. In this study, SLA was used as a positive control and run in parallel to all experiments along with unstimulated (UNS) culture condition. After 72 hrs, the supernatants were collected and centrifuged at 1000 g for 10 min. The quantitative yield of secreted IFN-γ was measured by BD OptiEIA (BD bioscience SD, USA) according to manufacturer’s protocol. Similarly, Granzyme B (GrB) levels in culture supernatants of three HLA A2 positive treated VL subjects were quantified with an ELISA assay (MABTECH AB).

### T cell proliferation assay

The isolated PBMCs ((2 × 10^6^ cells/well in RPMI-1640 complete medium) from 3 treated VL subjects were seeded in 24 well plate and incubated overnight at 37 °C and 5% CO_2_. The non-adherent cells were collected and stained with CFSE (Carboxyfluoresceinsuccinimidyl ester) dye (Biolegend, SD, USA) according to manufacturer’s protocol. Stained lymphocytes were placed back to the respective wells which contained adherent macrophages and stimulated with 20 μg/ml synthetic peptide for 96 hrs and kept in humidified condition as described earlier. The VSV8 peptide and SLA were run in parallel to each experiment and was used as negative control and positive control respectively. Culture supernatant containing T cells were harvested and washed twice in 2 ml stain buffer at 500 g for 10 min. The cells were then stained with anti-CD3- PerCP and anti-CD8- PE (BD Biosciences). The samples were acquired by Becton Dickinson FACS Calibur and analyzed by CellQuest software.

### Intracellular cytokine produced from CD8+ T cells against selected

To evaluate the ability of the selected epitopes to trigger the immune cells by CD8+ T cell dependent manner, PBMCs (1 × 10^6^/ml) from five HLAA2 positive VL treated subjects were incubated for overnight in the presence or absence of peptide at 37 °C and 5% CO_2_. Additionally, six HLA A2 negative VL treated subjects were also considered for this study. Stimulation with SLA was run in parallel to all experiments as described previously^50^. After overnight incubation followed by 6 h incubation with brefeldin-A (1 mg/ml), cells were harvested, washed with PBS, and stained with anti-CD3-PerCP (BD Biosciences) and anti-CD8-FITC(BD Biosciences) conjugated antibodies for 30 min at 4 °C. The cells were then washed with stain buffer, fixed and permeabilized Cytofix-Cytoperm buffer (BD Biosciences) for 20 minutes at 4 °C. The intracellular level of IFN-γ was stained with anti-IFN-γ PE-conjugated antibody (BD Biosciences) for 30 minutes and washed with perm wash buffer (BD Biosciences). A logical gate set using was used to measure the co-expression of intracellular CD8+ ve IFN-γ. At least 30 000 cells were acquired for each analysis, and the results are shown in percentage gated value (% gated).

### Statistical analysis

All data were expressed as mean ± SD (standard deviation). Significance was accessed by a student t-test and a value of P < 0.05 was considered to be significant. Statistical analysis was carried out using GraphPad Prism 5 software.

## Results

### Screening of potential HLA A0201 restricted epitopes

In a preselected environment, the Syfpeithi, Rankpep, Epijen, nHLApred web-server predicted the potential epitopes from the retrieved amino acid sequence of Leif and TSA. To improve the efficacy of epitope selection, we employed Trost *et al*. theory and selected the epitopes which were predicted by at least three web-servers. Based on the ranking scores, 9potential epitopes were selected for further analysis (Table 1). The cutoff value for each epitope prediction web-server was adopted from the published literature^17,51^. One of the major barriers in epitope-based vaccine development is the sequence homology with human proteomes. The peptides screened through this sieving process were further blasted against *Homo sapiens* (taxid: 9606), to exclude such epitopes with the ability for generating autoimmune responses, but most of them had shown sequence identity and query coverage <80%, respectively.

**Table 1:** Characteristics of computationally predicted *L*. *donovani* specific CD8+ T cell 9-mer epitopes.

Protein	Peptide	Position^a^	Syfpeithi^b^	Rankpep^c^	Epijen^d^	nHLApred^e^
Eif-2α	T L D H L L V L L	61–69	27	79	9.046	1
	V L L E K A T I L	67–75	26	87	9.027	1
	S L A R R K L L L	41–49	25	78	8.06	1
	L L L A E P F P V	47–55	24	67	9.978	1
	K V L T L F A E V	298–305	22	80	9.49	
TSA	M L A D K T K S I	104–112	25	92	8.99	1
	R L L E A F Q F V	158–166	24	88	9.57	0.99
	F I I D P N G M V	131–139	23	66	8.65	1
	S M D S E Y A H L	77–85	22	71	—	0.92

The consensus based approach was followed up to screen the potential epitopes.

^a^Amino acid position in the protein sequence.

^b^Threshold value set on 22.

^c^Threshold score set on 65.

^d^Threshold value set on 5%.

^e^Cutoffscore set on 0.5.

**Figure 1:**
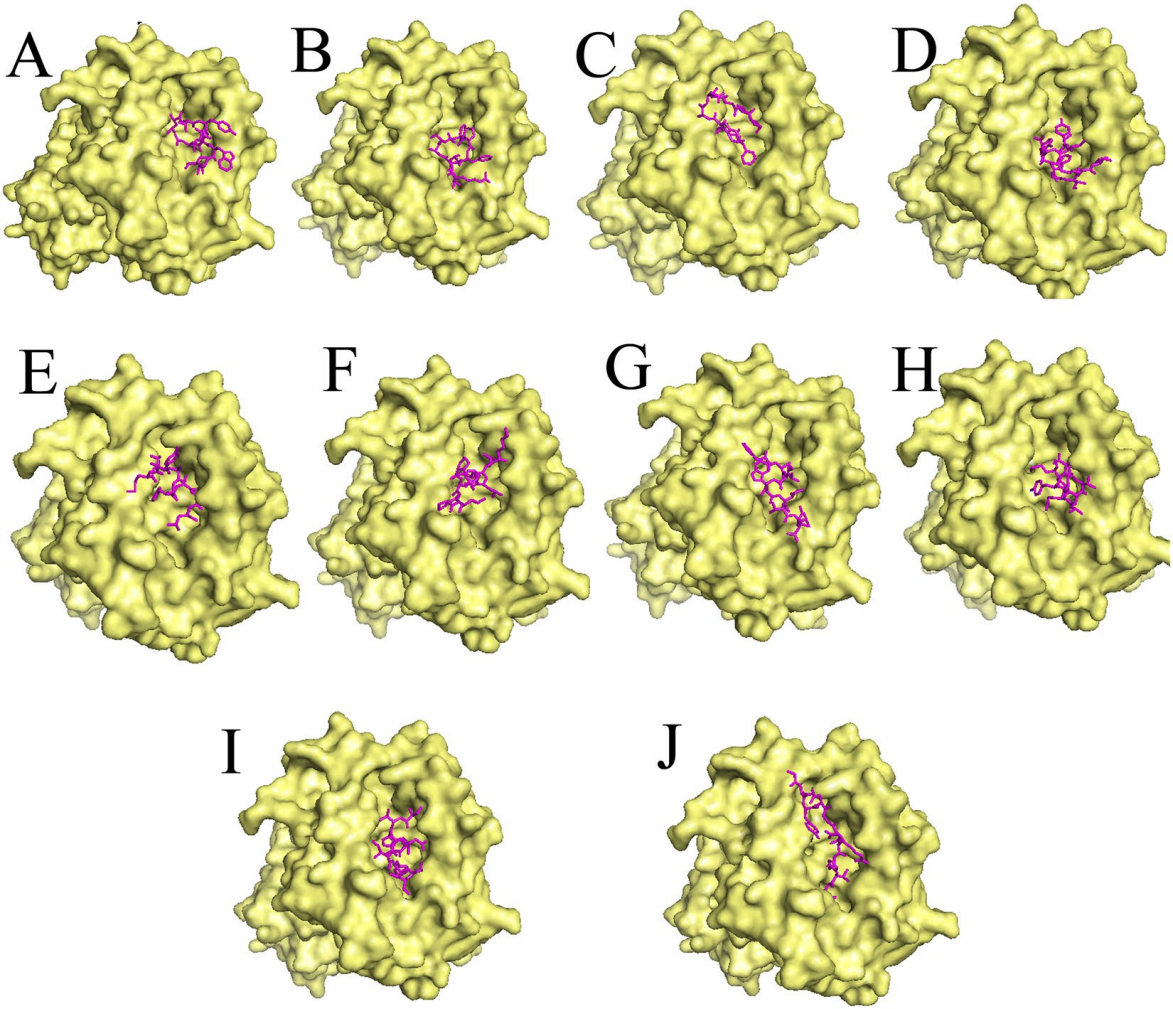
Docking of HLA A0201 allele with the selected epitope. Candidate peptides were predicted to locate onto the peptide-binding cleft of the HLA molecules by using fire dock web server. Front view of the docked conformation is shown where the epitopes (represents in magenta color) interact with the α chain of HLA-A 0201. Here, 10-mer peptide derived from tumour-specific antigenic peptide was used as positive control (**J**). Figure (**A**–**I**) represents the peptide P1-P9 respectively.

For the sake of ascertaining the proper binding orientation between the shortlisted epitopes and HLA A00201 molecule, docking calculation was used. A decamer peptide bound with HLA-A 0201(PDB ID: 1I4F) was retrieved from PDB database, the covalently bound peptide was separated and used as positive control. The binding orientation of each peptide to the α1 and α2 binding groove was predicted by PatchDock web-server. The results revealed that the anchor residues of all the selected peptides interact with in the α1 and α2 binding groove, which signify the favored mode of presentation to the T cell receptor (Fig. 1A–I). The Geometric shape complementarity scores for P1, P2, P3, P4, P5, P6, P7, P8 and P9 peptides were 6882, 7392, 6290, 6764, 7280, 7480, 7054, 7602 and 6434 respectively, which were very close to the binding score of the tumor-specific antigenic peptide (7998) (Table 2). In contrast, further refinement with FireDock web-server revealed an unstable interaction for P3 and P6 (binding energy (kcal) −21.77 and −27.25 respectively). Except P3 and P6, the binding energy of all the epitopes was bellow of −40 kCal which signifies the selected epitopes could be loaded preferentially by HLA-A 0201 allele.

**Table 2:** Diverse immunogenic properties such as TAP binding, Immunogenicity, HLA-Peptide binding affinity were predicted by different web-server.

Sl. No.	Peptide	TAPPred Score	Binding	Immunogenicity Score	Toxicity	Patch Dock	Fire Dock
P1	TLDHLLVLL	4.404	Intermediate	0.04687	Non-Toxin	6882	−44.05
P 2	VLLEKATIL	8.165	High	0.0345	Non-Toxin	7392	−37.64
P 3	SLARRKLLL	3.841	Inermediate	−0.10366	Non-Toxin	6290	−21.77
P 4	LLLAEPFPV	7.245	High	0.21515	Non-Toxin	6764	−44.29
P 5	KVLTLFAEV	7.908	High	0.22638	Non-Toxin	7280	−42.38
P 6	MLADKTKSI	7.509	High	−0.4171	Non-Toxin	7480	−27.25
P 7	RLLEAFQFV	8.485	High	0.21609	Non-Toxin	7054	−46.06
P 8	FIIDPNGMV	−0.40	Un detectable	−0.02537	Non-Toxin	7602	−48.37
P 9	SMDSEYAHL	7.589	High	−0.01333	Non-Toxin	6434	−42.56
Control	GVYDGREHTV	7.055	High	0.24875	Non-Toxin	7998	−65.11

Furthermore, the ability of peptide to affect the host cell was evaluated by ToxinPred.

### MD trajectory analysis

**Figure 2:**
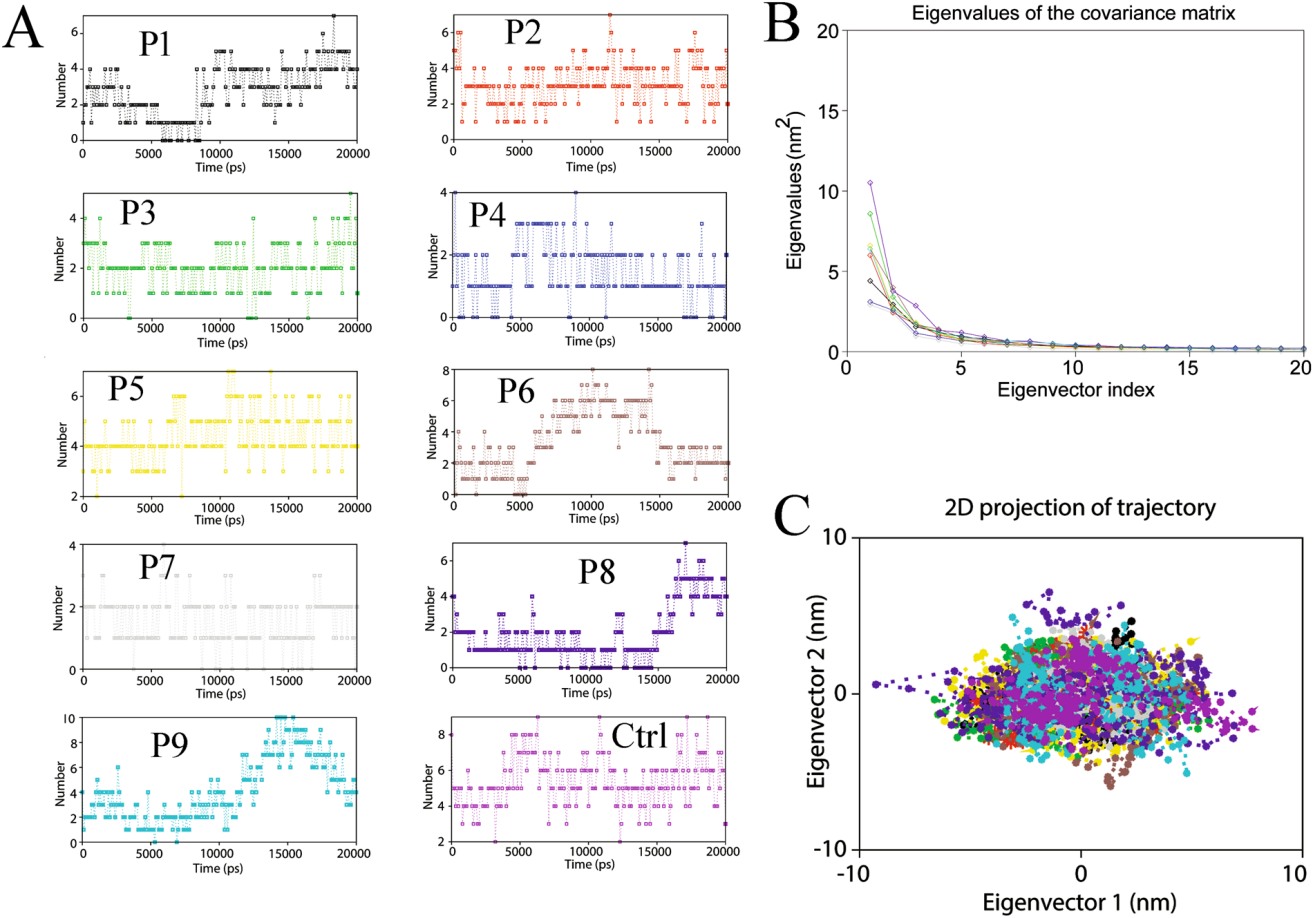
Binding stabilityofHLA-epitope complex was assessed by MD simulations. (**A**) Analysis of variation of intermolecular H-bond of HLA-epitope complxes during MD simulation. (**B**) Comparative analysis of eigenvalues plotted against the corresponding eigenvector indices derived from Cα covariance matrix during 20 ns MD simulations. (**C**) The 2D projection of each HLA-epitope complex over in phase space along the first two principal eigenvectors components during 20 ns MD.

In this study, MD simulations were employed to comprehend the stability and dynamics of the binding of epitopes (predicted peptides) to the active cavity of HLA alleles obtained from protein-peptide docking. To determine the stability and mechanistic aspects of the protein-peptide interactions RMSD, Rg and intermolecular H-bonds were computed. To access the dynamic stability of systems, a commonly used global measure of protein fluctuations i.e., RMSD was calculated. The dynamic stabilities (magnitude of fluctuations) of each complex was estimated using RMSD changes over 20 ns MD simulations as shown in Fig. S1. All the complexes showed a similar trend in RMSD i.e., average RMSD of ~2.19 Å indicates that all the systems attained stable conformations after equilibration. Though large RMSD fluctuations were observed in case of control complex during initial 10 ns, but was found be equilibrated with fewer RMSD fluctuations observed after 14 ns. The Rg which depict the compactness of the system, a property linked to the molecular volume and compactness, was to be found to be stable in all the systems except the control group (on the higher side). The average Rg of each system was found to be ~23.87 Å (Fig. S1). No such prominent deviation was observed in most of the systems, indicates that the minimal conformational changes of the complexes. To further confirm the stability of the protein-peptide systems, the DSSP algorithm was used to evaluate the changes in the secondary structure during MD simulations. No significant changes in structural elements (helical and β -sheet content remained constant) was observed during the MD simulations of each system. The stability of the docked HLA-epitope complexes was further investigated by using the *gmx hbond* utility, the intermolecular H-bonds formed between HLA and epitope was determined. Most of the cases, it was observed that the number of hydrogen bonds was not found to be constantly stable, which under underwent significant change during 20 ns MD as shown in Fig. 2A. It seems, few H-bonds at the docking level were found to be broken during MD simulation, later they were well compensated by van der Waals and hydrophobic contacts. Minute observation of each HLA-epitope complex during MD simulations revealed that most of the peptides kept their initial conformation during the simulation and tends bind in the binding groove of HLA active pockets.

### Essential dynamics

Proteins are apparently flexible that, upon binding to its receptors/ligands/peptides, display transition from one form to energetically favorable form to accomplish its functions. The local or global changes are pivotal to apprehend the complex formational modulations in proteins (David and Jacobs, 2014). Henceforth, PCA was performed on each system to examine the overall motion of the protein molecules i.e., to elucidate intrinsic flexibility. Diagonal covariance matrices were built over the Cα atoms of the complex trajectory. It has been found that the majority of protein dynamics can be successfully described by the first few eigenvectors or principal components of the entire system^52^. The eigen values obtained through the diagonalization of the covariance matrix elucidates the atomic contribution on the motion as shown in Fig. 2B. Similarly, the eigenvectors explain a collective motion accomplished by the particles. The spectrum of the corresponding eigenvalues indicated the level of fluctuation and dynamic behavior of protein molecule in the system and confined within the first two eigenvectors. Within the top eigenvectors, the first two accounted for a significant amount of overall motion. The projection of first two principal components displays the motion of the complex in phase space (Fig. 2C) where, the overall flexibility was calculated by the trace of the diagonalized covariance matrix. The trace values of covariance matrixes of the HLA-epitope complexes were found to be 19.69 nm^2^, 19.55 nm^2^, 24.08 nm^2^, 23.47 nm^2^, 20.81 nm^2^, 22.87 nm^2^, 14.97 nm^2^, 29.31 nm^2^, 23.93 nm^2^ and 21.99 nm^2^ respectively. From the analysis of trace of covariance matrix, it is clear that most of the systems occupied a small region of phase space showing least flexibility especially along the first principal component (PC1). The modevectors. py (a PyMOL script) was employed to generate porcupine plot depicting the graphical summary of the motions along the trajectory (Fig. S1C). The C-alpha atom has a cone pointing in the direction of the motion of the atom; the length of the cone reflects the amplitude of the motion and the size of the cone indicates the number of such C-alpha atoms. Most of the systems portrayed a uniform motion while the control systems displayed a different kind of motion, which was well supported by RMSD and Rg analysis.

### Estimation of binding free energy

Compared to various energy scoring functions executed in different molecular docking tools, binding free-energy calculation techniques i.e., MM/GBSA and MM/PBSA methodologies have shown better accuracy for binding energy ranking^53^. Henceforth, in this study, we selected 200 equal-interval snapshots between 10 ns and 20 ns of each simulation to estimate binding free energies for each HLA-epitope complex. Depending on the composition of amino acids (within peptides), different interactions ranging from hydrophobic, hydrogen, electrostatics and pi-pi interactions are formed between the peptide and the distinct HLAs. Each of these individual interactions contributes either positively or negatively to the overall binding free energy. The different energy terms contributing to the binding free energy of each HLA-epitope complex has been summarized in Table 3. The estimated binding energies for each of these complexes were within the range of −45.39 ± 3.64 to −137.17 ± 5.76 kJ/mol. As evident from Table 3, it can be observed that in most of the systems, polar solvation energies (PSE) opposed binding of epitopes while van der Waals (vdW), electrostatic forces and solvent accessible surface area (SASA) energy favored the binding process. Principally, among the various energy terms, van der Waals and electrostatic energy contribute the maximum to the free energy of binding which was followed by SASA energy.

**Table 3:** MM/PBSA binding free energies of HLA-epitope complexes.

Complexes	Van-der Waal energy (kJ/mol)	Electrostatic energy (kJ/mol)	Polar solvation energy (kJ/mol)	SASA energy (kJ/mol)	Binding energy (kJ/mol)
P1	−194.864 ± 2.003	−289.547 ± 5.927	451.081 ± 5.681	−22.049 ± 0.208	−55.759 ± 3.529
P2	−169.421 ± 2.417	−264.894 ± 4.216	411.390 ± 5.922	−22.412 ± 0.199	−45.393 ± 3.643
P3	−151.158 ± 2.325	−391.484 ± 7.157	478.777 ± 7.731	−20.186 ± 0.234	−84.100 ± 5.727
P4	−177.737 ± 5.195	−73.809 ± 3.137	225.340 ± 6.335	−22.406 ± 0.607	−48.895 ± 3.470
P5	−226.467 ± 2.364	−277.234 ± 2.679	472.315 ± 5.179	−27.224 ± 0.271	−58.536 ± 3.644
P6	−80.366 ± 2.234	−399.525 ± 8.577	357.409 ± 5.465	−15.559 ± 0.269	−137.172 ± 5.762
P7	−178.236 ± 1.761	−86.106 ± 4.037	236.282 ± 5.629	−22.319 ± 0.158	−50.468 ± 2.370
P8	−235.589 ± 2.495	−270.829 ± 8.648	483.259 ± 8.306	−31.399 ± 0.190	−54.558 ± 3.213
P9	−151.049 ± 2.331	−391.916 ± 6.894	478.524 ± 7.719	−20.193 ± 0.233	−84.281 ± 5.890
Control	−114.395 ± 1.857	−235.831 ± 6.319	287.102 ± 8.985	−15.703 ± 0.285	−78.497 ± 7.050

### Evaluation of vaccine potential of selected epitopes

A peptide with a proteasomal recognition site is not an ideal vaccine candidate because it will be degraded during antigen processing. The identification proteasomal cleavage site using PAProC.22 revealed that no selected epitopes have proteasomal recognition site (Data not shown). Further analysis also revealed that except P8, all the epitopes have TAP binding preferences which may preferentially transport from cytosol to endoplasmic reticulum to be presented on cell surface (Table 2). Although computational algorithms have improved to predict the binding affinity between peptide/HLA complexes or TAP binding affinity, they have not been trained to predict immunogenicity. The major limitation persists using such prediction algorithms is the presence of peptides with predicted high-affinity scores that will never lead to evoke an immune response^54^. The peptide with a high immunogenicity score predicted by T cell class I pMHC immunogenicity predictor was supposed to have a high potentiality to activate the cellular immunity. Our data showed that the epitopes immunogenicity scores ranged from −0.4171 to 0.22638 (Table 2). Although the precise role of CD8+ T cells during primary immune responses is controversial, these cells play a prominent role in protecting mice from a secondary challenge^9,55^. Furthermore, a good vaccine candidate should have the ability to modulate a specific immune cell activation that can targets the infected cells. To corroborate that ability, toxicity prediction was employed which depicted that all the nine shortlisted epitopes are non-toxic in nature (Table 2). All these data suggest the antigenic and immunogenic potential of the shortlisted epitopes and kept for further analysis.

### Population coverage and conservancy analysis

The human hosts are genetically heterogeneous and express a different set of HLA. Due to this heterogeneity, different HLA may have different specificities with diverse T-cell epitope repertoires. Therefore, the NetMHC web server was used to acquire HLA alleles with high epitope-binding force and to monitor the HLA specificity. The data obtained from this study revealed that the selected set of epitopes showed extensive binding affinity to the 46 diverse HLA class-I allele (Table 4). Further analysis revealed that except P3, all the selected epitopes are likely to respond more than 50% of genetically heterogeneous human populations (Table 4).

**Table 4:** HLA cross presentation ability and theoretical population coverage of selected peptides predicted by NetMHCPan and IEDB web-server respectively.

SL. No.	Peptide	Cross Presenting HLA Allele	No of Allele	Theoretical population coverage	
1	TLDHLLVLL	HLA-A0101, HLA-A0201, HLA-A0202 HLA-A0205, HLA-A0206, HLA-A0207 HLA-A0211 HLA-A0212 HLA-A0216 HLA-A0217 HLA-A0219 HLA-B3901 HLA-B4801 HLA-C0401 HLA-C0501 HLA-C0802	16	71.02%	**88**.**10%**
2	VLLEKATIL	HLA-A0201, HLA-A0211 HLA-A0212 HLA-A0216 HLA-A0217 HLA-A0219 HLA-A0250 HLA-A3201 HLA-B0801 HLA-B0802 HLA-C0401 HLA-C0602 HLA-C0701	13	74.32%	
3	SLARRKLLL	HLA-A0202 HLA-A0217 HLA-A8001 HLA-B0801 HLA-B0802 HLA-B0803 HLA-B1402 HLA-B4013 HLA-B8301 HLA-C0701	10	30.97%	
4	LLLAEPFPV	HLA-A0201, HLA-A0202, HLA-A0203, HLA-A0205 HLA-A0206 HLA-A0207 HLA-A0211 HLA-A0212 HLA-A0216 HLA-A0217 HLA-A0219 HLA-A0250 HLA-A3201 HLA-A3207 HLA-A3215 HLA-A6901 HLA-B4013 HLA-C0802	19	50.69%	
5	KVLTLFAEV	HLA-A0201, HLA-A0203, HLA-A0205 HLA-A0206 HLA-A0211 HLA-A0212 HLA-A0216 HLA-A0219 HLA-A0250 HLA-A3001 HLA-A3201 HLA-A3207 HLA-A6901 HLA-C1502	14	50.01%	
6	MLADKTKSI	HLA-A0201, HLA-A0202, HLA-A0203 HLA-A0205 HLA-A0211 HLA-A0212 HLA-A0216 HLA-A0217 HLA-A0219 HLA-A0250 HLA-A3201 HLA-A3215 HLA-A6901 HLA-B0801 HLA-B0802 HLA-B0803 HLA-B1402 HLA-B1502 HLA-B8301 HLA-C0303 HLA-C0602 HLA-C0701 HLA-C1203 HLA-C1402	24	77.00%	**91**.**90%**
7	RLLEAFQFV	HLA-A0201, HLA-A0202, HLA-A0203 HLA-A0205 HLA-A0206 HLA-A0207 HLA-A0211 HLA-A0212 HLA-A0216 HLA-A0217 HLA-A0219 HLA-A0250 HLA-A3001 HLA-A3201 HLA-A3207 HLA-A6901 HLA-B0803 HLA-B2720 HLA-B4013 HLA-C0602 HLA-C1502	21	60.30%	
8	FIIDPNGMV	HLA-A0201, HLA-A0202, HLA-A0203 HLA-A0205HLA-A0206 HLA-A0207 HLA-A0211 HLA-A0212 HLA-A0216 HLA-A0217 HLA-A0219 HLA-A0250 HLA-A2501 HLA-A2601 HLA-A2602HLA-A2603 HLA-A6802 HLA-A6901 HLA-B4601 HLA-C0303 HLA-C0501 HLA-C0602 HLA-C0802 HLA-C1203 HLA-C1502	25	74.99%	
9	SMDSEYAHL	HLA-A0201, HLA-A0202 HLA-A0211 HLA-A0212 HLA-A0216 HLA-A0217 HLA-A0219 HLA-A0250 HLA-A6901 HLA-B0802 HLA-B0803 HLA-B1503 HLA-B3503 HLA-B3901 HLA-B4801 HLA-C0401 HLA-C0501 HLA-C0802 HLA-C1402	19	63.70%	

Total Theoretical Population Coverage 94.16%.

The key amino acid residues with essentially lower variability under immune pressure are supposed to play a crucial role in peptide-based vaccine design^56^. In this study, epitope conservancy analysis recommended a high degree of conservancy of the shortlisted epitopes across the different *Leishmania* species. Though the level of conservancy of the shortlisted epitopes varies from 78% to 100%, four epitopes, namely P1, P2, P3 and P5 was found to be the most conserved epitope, which shared 100% sequence homology with *L*. *infantum*, *L*. *major*, *L*. *braziliensis*, *L*. *Mexicana and L*. *Guyanensis* (Table 5). Interestingly, the set of epitopes are highly conserved (100%) in *L*. *donovani and L*. *infantum* and moderately conserved (>89%) among other *Leishmania* species (except P4) (Table 5). Thus, the outcomes indicate that the selected epitopes might be a universal candidate to provoke an efficient epitope-based immune-activation against multiple *Leishmania* species.

**Table 5:** Epitope conservancy among different *Leishmania* species was analyzed by epitope conservancy tool available at IEDB web-server and BLASTP analysis.

Peptide	*L*. *donovani*	*L*. *infantum*	*L*. *L*. *majoror*	*L*. *braziliensis*	*L*. *mexicana*	*L*. *guyanensis*
T L D H L L V L L	100%	100%	100%	100%	100%	100%
V L L E K A T I L	100%	100%	100%	100%	100%	100%
S L A R R K L L L	100%	100%	100%	100%	100%	100%
L L L A E P F P V	100%	100%	100%	78%	100%	78%
K V L T L F A E V	100%	100%	100%	100%	100%	100%
M L A D K T K S I	100%	100%	100%	89%	100%	89%
R L L E A F Q F V	100%	100%	100%	89%	100%	100%
F I I D P N G M V	100%	100%	88%*	86%**	86%**	86%**
S M D S E Y A H L	100%	100%	89%	89%	100%	100%

*Query Coverage is 88%, **Query Coverage is 77%.

### HLA stabilization assay

We then employed the simplest ways to measure the efficiency of peptide binding stability with T2 cell line. Without efficient TAP-mediated transport, T2 cells are defective for an endogenous class I presentation. When T2 cells co-culture with exogenous peptide are capable to bind class I allele and stabilize the expression of HLA on the cell surface^57^. As shown in Fig. S2, P1 (TLDHLLVLL), P2 (VLLEKATIL), P4 (LLLAEPFPV), P5 (KVLTLFAEV) and P7 (RLLEAFQFV) were strongly bound to the HLA-A*0201 molecule on T2 cells, whereas other epitopes such asP3 (SLARRKLLL), P6 (MLADKTKSI), P8 (FIIDPNGMV) and P9 (SMDSEYAHL)were relatively weakly bound to the HLA-A*0201 molecule. Specifically, the relative FI value for P1, P2 and P5 and P7 was above 2. Peptides, P4 also revealed a good binding affinity with FI value 1.81 (Fig. S2). Therefore, these peptides were selected for immunogenicity studies in treated VL subjects.

**Figure 3:**
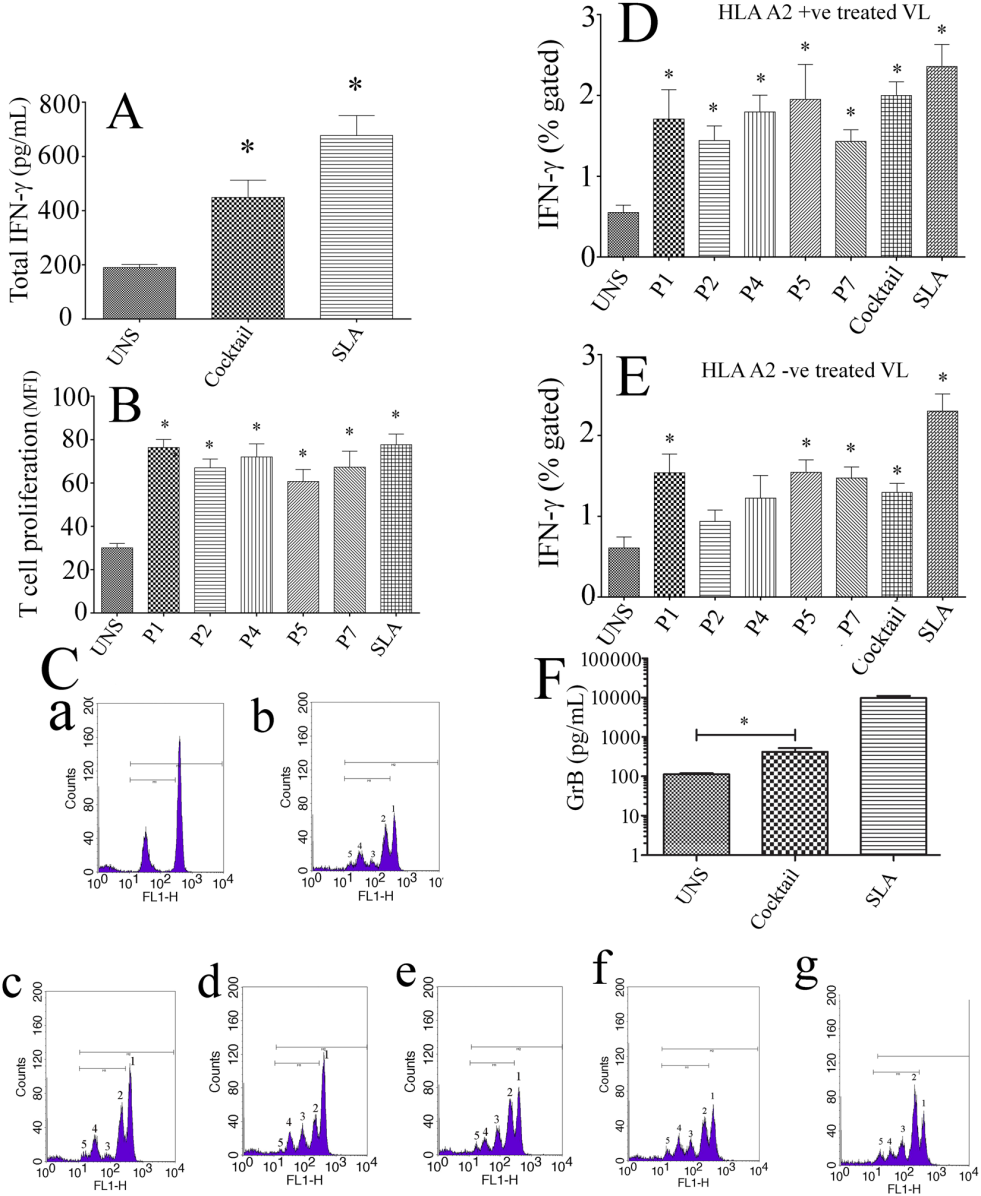
The ability of the optimal set of epitopes to modulate the host immune system was assessed in treated VL subjects. (**A**) The ELISA assay showed that challenge with the cocktail of epitopes produced total IFN-γ significantly as compared to unstimulated culture condition. (**B**) The mean Fluorescent Intensity (MFI) of the proliferated regions (M1) was assessed. The result showed that the PBMCs of treated VL subjects when stimulated with peptide proliferated CD8+ T cell significantly as compared to unstimulated culture condition. (**C**) Representative FACS plot showing CD8+ T cell proliferated significantly up to 5 generation against the epitopes in treated VL subjects. Here, b, c, d, e, f represents P1, P2, P4, P5 and P7 respectively. P1 (a) was used as unstimulated culture condition where as SLA (g) was used as positive control. (**D**) Challenge with peptide either individually or as cocktail modulates the protective immune response by inducing CD8+ IFN-γ in HLA A2+ ve cured VL subjects. (**E**) The epitopes P1, P4 and P5 as well as the cocktail of optimal set of epitopes triggered the HLA A2-ve host immune cells by producing CD8+ IFN-γ. Here, soluble Leishmania antigen (SLA) was used as a positive control. (**F**) Stimulation with peptide cocktail induces a significant level of GrB in PBMCs of treated VL subjects as compared to unstimulated culture condition.

To evaluate the epitope influence on T cell proliferation, T cell proliferation assays were carried out using the PBMCs isolated from three VL treated subjects. As expected, the epitopes with high FI values proliferated the CD3+ ve CD8+ ve T lymphocytes significantly as compared to unstimulated and negative control (Fig. 3B). More precisely, PBMCs cultured with the epitopes triggered the sensitized T cells up to 5 generation indicating their vaccine potential^17,51^ (Fig. 3C).

### Protective response against the peptide in VL treated subjects

Initially, the ability of an optimal set of epitopes to evoke the immune cell to produce IFN-γ was determined by ELISA (Fig. 3A). The PBMCs of treated VL cases when stimulated with the cocktail of epitopes as a source of antigen, showed significantly increased secreted levels of IFN-γ. To ensure the source of IFN-γ, intracellular (CD3+ ve CD8+ ve) level of IFN-γ was observed in both HLA A2 positive and negative VL treated subjects (Fig. 3D,E). As expected, the PBMCs of HLA A2 positive treated VL subjects stimulated with the optimal set of epitopes modulates CD3+ ve CD8+ ve cells to produce intracellular IFN-γ significantly as compared to unstimulated culture condition. Interestingly, against HLA A2 negative treated VL subjects, some of the epitopes (P1, P5 andP7), as well as a cocktail triggered CD3+ ve CD8+ ve IFN-γ level (Fig. 3E). Similarly, as shown in Fig. 3F, the cocktail of immunogenic peptides induces a significant level of GrB in culture supernatants from immune individuals as compared to unstimulated culture condition. All these results indicated that these optimal set of epitopes were capable to evoke the desired immune response with wide population coverage.

## Discussion

So far, a range of methodologies has been implemented to address the need for vaccine candidates that are efficient against leishmaniasis. Due to the conventional approaches, most of the vaccine candidates became unsuccessful because of several safety issues, poor antigen response, a lack of good animal models, and a lack of standardization. In recent years, reverse vaccinology approach was widely implemented to design subunit vaccines and epitope-based immunotherapy. In a similar node, two potent antigens namely Leif and TSA, when used with MPL-SE adjuvant have shown an enhanced IFN-γ production. Though several authors indicated the vaccine potential of these antigens^19,28,58^, without adjuvant, they failed to generate a cell-mediated immune response^59,60^. The recent study affirmed the subtle variations in antigen presentations as one of the reasonable explanations for the failure of a vaccine candidate^61^. Thus, the present study is focused on polytope vaccines because it contains specific immunogenic components of the pathogens and can generate a desired immune response.

Analysis of Leif and TSA sequence on SYFPEITHI, Rankpep, Epijen, nHLApred and SVMHC software indicated the presence of nine high scored HLA A0201 binding epitopes. We have adopted a higher threshold value because *in vitro* validation is not so easy. As the prediction is based on Trost *et al*. theory, therefore it can be easily speculated that a considerable proportion as high as 85% of epitopes have the ability to modulate the immune cells. BLASTP analysis also revealed that the consensus epitopes derived from Leif and TSA did not mimic any similarity with any of the proteins from the human host. This supports the argument that the selected potential hotspots in the form of consensus epitopes are ideal antigenic targets and guarantees a high specificity with low cross-reactivity.

The efficiency of the TAP-mediated transportation of HLA restricted epitopes is mostly relying on its TAP binding affinity. The TAPPred analysis revealed that all the shortlisted peptide (except P8) have either a high or an intermediate TAP binding affinity which is an important step in endogenous antigen processing (Table 2). These result suggested a valid and effective trans-migration chance of promiscuous epitopes into the endoplasmic reticulum where it can bind to MHC class I allele.

Predicting the binding orientation of epitope with HLA molecules is the single most selective of the many events leading to antigen presentation. In this notion, the results obtained from Patchdock and fire dock web-server suggested that the selected epitopes bind between α1 and α2 binding groove with sufficient affinity and stability which allows the translocation of intact epitope–HLA complexes to the cell surface. Fascinatingly, the epitopes generated by the antigen processing machinery may bind to more than one HLA allele^62^. This cross-presentation property of epitopes has allowed us to categorize the HLA alleles that present the shared epitope, which could lead to the generation of a multi-epitope universal vaccine. Here we discovered nine novel epitopes which can plausibly be presented by 45 other HLA class-I alleles than HLA-A0201, thereby broadening the target populations up to 94.16%. Investigation in this regard affirmed that the epitopes such as P1, P2, P6 and P8 derived from Leif and TSA shared more than 70% of population coverage individually. Further analysis by Toxinpred depicted that all nine of the selected epitopes are safe to the host tissue.

T-cell epitope discovery is complicated by the co-dominance and polymorphism of HLA alleles and antigens diversity^63^. This is of reasonable significance since the reckoning of such epitopes conserved among the varied *Leishmania* species could plausibly improve its applicability. Specifically, most of the epitopes that stabilized the HLA A2 on cell surface shared 100% sequence homology with other *Leishmania* species. Given the low phylogenetic diversity, it is apparent that the same set of epitopes could evoke a common pattern of protective immune response^51^.

A close association between stability and immunogenicity was established^64^. Several authors have reported that a more stable peptide-HLA complex correlates with higher immunogenicity^65–68^. HLA stabilization assay confirmed that five epitopes (P1, P2, P4, P5 and P7) remain associated with HLA A2 allele on the surface of T2 cell line for long enough for recognition of circulatingCD8+ CTLs. Interestingly, the measured fluorescent index (FI) derived from HLA stabilization assay exhibited a modest correlation with the positive immunogenicity score predicted by class I pMHC immunogenicity predictor. The findings indicated that five nonamers, P1, P2, P4, P5 and P7, resulted more than ~1.9 fold increase in the FI values have positive predicted immunogenicity score. Additionally, the presence of main anchor residue such as Leu or Val at position 2/9 enhances its reliability as a valid and effective combination for a successful vaccine candidate^69^. A support for this notion came from ELISA assay where epitope either individually or as a cocktail-induced IFN-γ production significantly in treated VL subject. It can be convincingly contemplated that those who had cured from VL developed a specific Th1 response against these five nona-peptides, indicating that it might promote pMHC-TCR binding during VL infection. Further, *in vitro* stimulation with these peptides individually against HLA A2 positive VL treated subjects depicted an enhanced CD3+ CD8+ T IFN-γ level compared to unstimulated culture condition. The major source of IFN-γ production from CD3+ CD8+ T cell implied its pro-active role against the optimal set of epitopes (P1, P2, P4, P5 and P7). Interestingly, stimulation with P1, P5 and P7 against HLA A2 negative treated VL subjects resulted an enhanced CD3+ CD8+ IFN-γ implying that irrespective of HLA diversity, the final set of epitopes are endowed with the ability to generate effector memory cells. PBMCs stimulated with these peptides resulted in a high degree of proliferating T cells (Fig. 3C(B–F)). The proliferation of T cell up to five generation strengthens the notion that epitope-specific memory repertoire eventually participated in the response. A support in this notion came from GrB analysis where a significantly up-regulated GrB level was observed against the peptide cocktail. Similar results against diverse peptide have been reported by Naouar and collaborators where they observed a higher level of GrB against the PBMCs of HLA A2 positive individuals^70^. Therefore, it can be persuasively speculated about the pro-active role of CD8+ T cell in the GrB production against the selected epitopes. Altogether, our analysis yielded five protective CD8 restricted epitopes that are nontoxic, immunogenic, conserved among *Leishmania* species and can induce an elevated level of IFN-γ in treated VL subjects which is a prerequisite of a vaccine candidate that controls leishmaniasis. However, a systematic investigation of these epitopes in humanized mouse models is desired to explore their ability as a polytope-based vaccine candidate.

## Electronic supplementary material

Supplementary information
