# Supplementary material for: The potential HLA Class I-restricted epitopes derived from LeIF and TSA of Leishmania donovani evoke anti-leishmania CD8+ T lymphocyte response
Source: Sci Rep. 2018 Sep 21;8:14175. doi: 10.1038/s41598-018-32040-x (PMC6154976; doi:10.1038/s41598-018-32040-x)
Supplement: Supplementary file 1 — Supplementary information [file 41598_2018_32040_MOESM1_ESM.pdf]

The potential HLA Class I-restricted epitopes derived from LeIF and TSA of *Leishmania donovani* evoke anti-leishmania CD8+ T lymphocyte response

Manas Ranjan Dikhit[1,2],Sushmita Das[3], Vijaya mahentesh[2],Akhilesh Kumar[2],Ashish Kumar Singh[2]Budheswar Dehury[4], Ajaya Kumar Rout[5], Vahab Ali[6], Ganesh Chandra Sahoo[1] ,Roshan Kamal Topno[7], Krishna Pandey[8], VNR Das[8], Sanjiva Bimal[2,*], Pradeep Das[9,*]

[1] BioMedical Informatics Division, Rajendra Memorial Research Institute of Medical Sciences, Agamkuan, Patna, 800007, Bihar, India
[2]Dept. of Immunology, Rajendra Memorial Research Institute of Medical Sciences, Agamkuan, Patna, 800007, Bihar, India
[3] Dept. of Microbiology, All India Institute of Medical Sciences, Patna, 801507, Bihar, India
[4] BioMedical Informatics Centre, ICMR-Regional Medical Research Centre, Bhubaneswar-751023, Odisha, India
[5] Biotechnology Laboratory, ICAR-Central Inland Fisheries Research Institute, Barrackpore, Kolkata-700120, West Bengal, India
[6] Dept. of Clinical Biochemistry, Rajendra Memorial Research Institute of Medical Sciences, Agamkuan, Patna, 800007, Bihar, India
[7] Dept. of Epidemiology, Rajendra Memorial Research Institute of Medical Sciences, Agamkuan, Patna, 800007, Bihar, India
[8] Dept. of Clinical Medicine, Rajendra Memorial Research Institute of Medical Sciences, Agamkuan, Patna, 800007, Bihar, India
[9] Dept. of Molecular Parasitology, Rajendra Memorial Research Institute of Medical Sciences, Agamkuan, Patna, 800007, Bihar, India

* Corresponding author

Dr. Pradeep Das
Scientist G, Director
Department of Molecular Parasitology,
Rajendra Memorial Research Institute of Medical Sciences
Agamkuan, Patna 800007, India.
E-mail: drpradeep.das@gmail.com

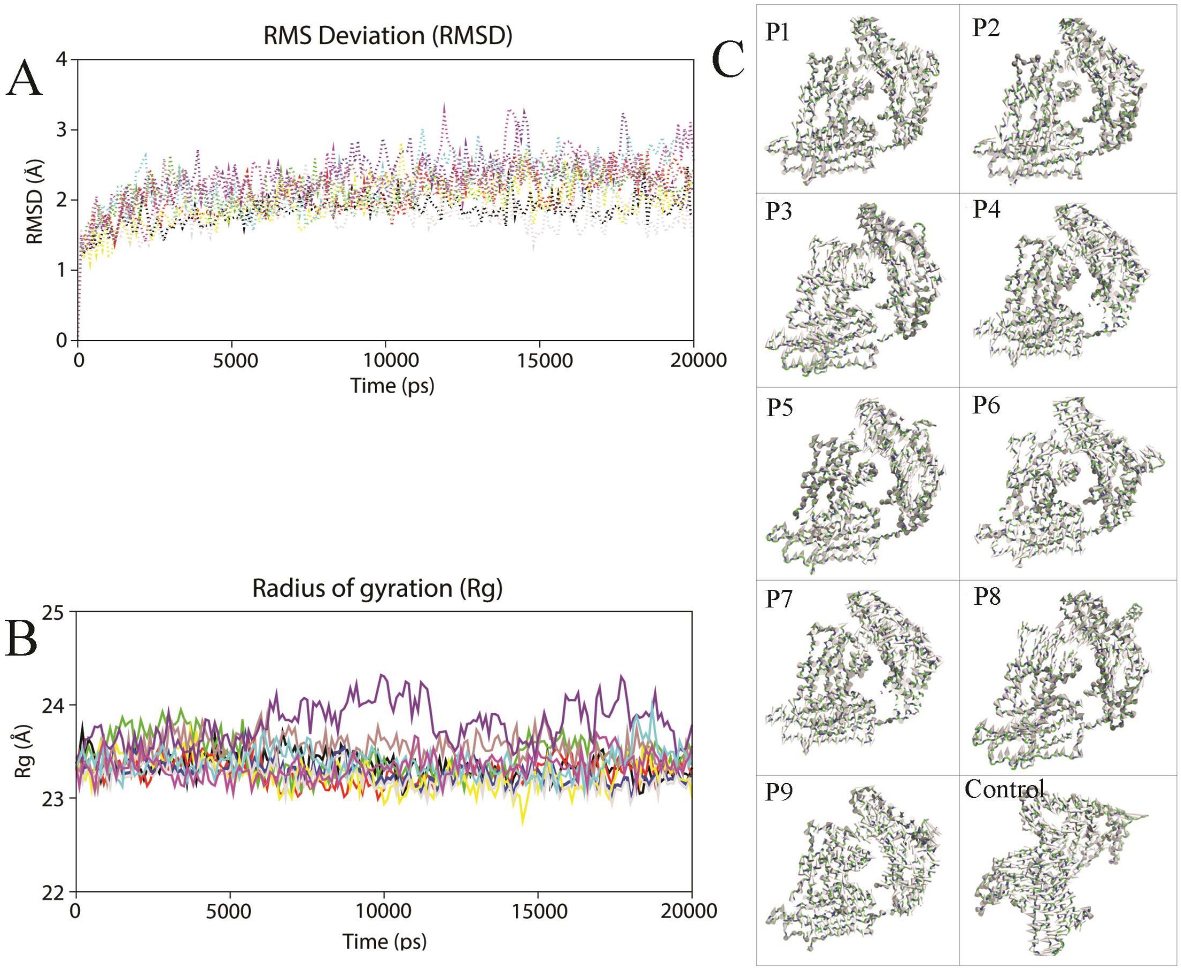

Fig.S1. MD simulations were carried out in GROMACS v5.0 (A)Time dependences of root mean square deviation (RMSD) profile of backbone atoms of HLA-epitope complexes during 20 ns MD simulations (Black, Red, Green, Blue, Yellow, Brown, Grey, Violet, Cyan and Magenta). (B) The conformational analysis of HLA-epitope complexes displaying the radius of gyration over 20 ns MD simulations. (C) Porcupine plot of PCA dominant motion HLA-epitope complexes. The first eigenvector represented in porcupine plot for HLA-epitope complexes.

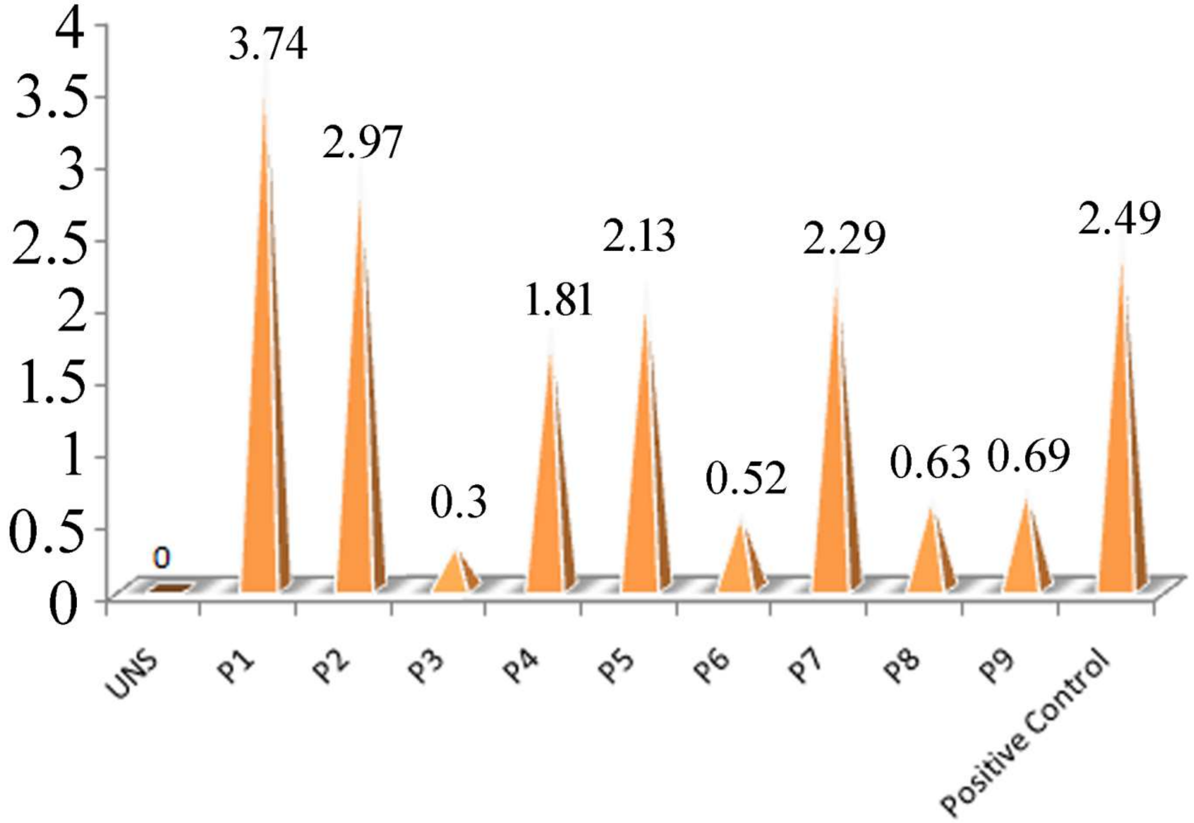

Fig.S2. Comparison of HLA-A02-epitope binding stability of the MHC-I complexes on the surface of T2 cell line. The fluorescent index (FI) was calculated and the FI value above 1 was considered as strong binding peptide. The referred mean fluorescent intensity of against the stimulated peptide and the data revealed that P1, P2, P4, P5 and P7 stabilized the HLA A2 as compared to other peptides. Here, Tumor specific peptide was considered as positive control.
